# Plasma Glutathione Levels Decreased with Cognitive Decline among People with Mild Cognitive Impairment (MCI): A Two-Year Prospective Study

**DOI:** 10.3390/antiox10111839

**Published:** 2021-11-19

**Authors:** Chieh-Hsin Lin, Hsien-Yuan Lane

**Affiliations:** 1 Department of Psychiatry, Kaohsiung Chang Gung Memorial Hospital, Chang Gung University College of Medicine, Kaohsiung 83301, Taiwan; simone36@cgmh.org.tw; 2 School of Medicine, Chang Gung University, Taoyuan 33302, Taiwan; 3 Graduate Institute of Biomedical Sciences, China Medical University, Taichung 40402, Taiwan; 4 Department of Psychiatry & Brain Disease Research Center, China Medical University Hospital, Taichung 40402, Taiwan; 5 Department of Psychology, College of Medical and Health Sciences, Asia University, Taichung 41354, Taiwan

**Keywords:** glutathione (GSH), mild cognitive impairment (MCI), Alzheimer’s disease (AD), cognitive function, biomarker

## Abstract

Glutathione (GSH) is a major endogenous antioxidant. Several studies have shown GSH redox imbalance and altered GSH levels in Alzheimer’s disease (AD) patients. Early detection is crucial for the outcome of AD. However, whether GSH can serve as a biomarker during the very early-phase of AD, such as mild cognitive impairment (MCI), remains unknown. The current prospective study aimed to examine the longitudinal change in plasma GSH concentration and its influence on cognitive decline in MCI. Overall, 49 patients with MCI and 16 healthy individuals were recruited. Plasma GSH levels and cognitive function, measured by the Mini-Mental Status Examination (MMSE) and Alzheimer’s disease assessment scale-cognitive subscale (ADAS-cog), were monitored every 6 months. We employed multiple regressions to examine the role of GSH level in cognitive decline in the 2 years period. The MCI patients showed significant decline in plasma GSH levels and cognitive function from baseline to endpoint (month 24). In comparison, the healthy individuals’ GSH concentration and cognitive function did not change significantly. Further, both GSH level at baseline and GSH level change from baseline to endpoint significantly influenced cognitive decline among the MCI patients. To our knowledge, this is the first study to demonstrate that both plasma GSH levels and cognitive function declined 2 years later among the MCI patients in a prospective manner. If replicated by future studies, blood GSH concentration may be regarded as a biomarker for monitoring cognitive change in MCI.

## 1. Introduction

Alzheimer’s disease (AD) is the most common cause of dementia; further, the number of patients with AD has been rapidly rising globally [1]. There have been several AD hypotheses including abnormal oxidative stress [2,3,4]; however, clinical trials aiming to reduce oxidative stress remain unsuccessful for dementia patients [5,6]. In order to recognize dementia in its earliest clinical stage, the concept of mild cognitive impairment (MCI), a slight cognitive impairment, was developed [7]. MCI, especially amnestic MCI (aMCI), is a risk factor or a prodromal stage of AD [8,9]. Of note, early intervention, for example, in the MCI stage, is crucial for the future control of this neurodegenerative disease, because the brain changes in pathology are found years or even decades before the cognitive and functional decline in AD patients [8,10,11,12]. Therefore, it is imperative to discover clinically feasible biomarkers that can predict or monitor the outcome or course of MCI [11,13].

Glutathione (GSH), a major endogenous antioxidant present in all mammalian cells and peripheral blood, plays a vital role in protecting neuronal cells from oxidative stress [14,15]. GSH scavenges harmful reactive oxygen species that are generated during different cellular/molecular processes including neurodegeneration [16]. 

Several studies have shown GSH redox imbalance in AD and decreased GSH levels in AD animal models as well as in the brain and blood of AD patients [15,17,18,19]. Though not all AD patients showed different GSH levels with normal controls [20,21], lower GSH concentration was associated with severer cognitive impairment among AD patients [20]. In addition, in non-demented people, higher plasma GSH levels had a lower risk of developing future AD and a better preservation of executive functioning longitudinally [22]. However, among patients with very early-phase AD, such as MCI, the temporal change in GSH levels and its influence on cognitive function remains unclear. This 2 year prospective study aimed to examine whether plasma GSH levels changed with cognitive decline in people with MCI.

## 2. Materials and Methods

### 2.1. Participants

Patients and healthy controls were screened and enrolled from Kaohsiung Chang Gung Memorial Hospital, Kaohsiung, which is a major medical center in Taiwan. The study was approved by the institutional review board (IRB) of the hospital and conducted in accordance with the current revision of the Declaration of Helsinki. All participants (both patients and healthy individuals) were evaluated by research psychiatrists after a thorough medical workup.

Participants were enrolled into this study if they (a) were ethnic Han Chinese, aged 50–100 years; (b) agreed to participate in the study and provided informed consent; (c) were physically healthy and had normal laboratory assessments (including blood routine and biochemical tests); (d) had sufficient education to communicate effectively and ability to complete the assessments of the study. 

We excluded participants if they had the following: (a) a history of significant cerebrovascular disease; (b) Hachinski ischemic score > 4; (c) major neurological, psychiatric, or medical conditions other than MCI; (d) delusion, hallucination, or delirium symptoms; (e) substance (including alcohol) abuse or dependence; (f) severe visual or hearing loss; (g) inability to follow the study protocol.

Patients with MCI fulfilled the National Institute of Neurological and Communicative Disorders and Stroke and the Alzheimer’s Disease and Related Disorders Association (NINCDS–ADRDA) criteria for aMCI [23] of a presumably degenerative nature defined as subjective memory complaint corroborated by an informant and insufficient global cognitive and functional impairment [24], and had a clinical dementia rating (CDR) [25] score of 0.5. 

Healthy individuals had a CDR score of 0.

All participants recruited were fully informed of all the procedures performed during the study and gave their written informed consent.

### 2.2. Cognitive Function Assessments

We used the Alzheimer’s disease assessment scale-cognitive subscale (ADAS-cog) [26] and the Mini-Mental State Examination (MMSE) [27] to evaluate cognitive function at months 0, 6, 12, 18, and 24. Year 2 (months 24) was the primary endpoint.

The MMSE is a commonly used cognitive test to screen and measure cognitive impairment in older people [28]. Its scores range from 0 (worst) to 30 (best).

The ADAS-cog is a popular cognitive assessment instrument used in cognitive aging studies. It consists of 11 tasks including word recall, naming, commands, constructional praxis, ideational praxis, orientation, word recognition, instructions remembering, spoken language ability, word-finding difficulty, and comprehension. Its scores range from 0 (best) to 70 (worst).

### 2.3. GSH Measurement

GSH concentration in plasma was measured at months 0, 6, 12, 18, and 24 using commercially available assay kits according to the manufacturer’s recommended protocol (Cayman, Ann Arbor, MI, USA). Briefly, 50 μL standard or plasma sample was added to each well of a 96-well plate and covered with the plate cover. The assay cocktail was prepared by mixing 11.25 mL MES Buffer, 0.45 mL reconstituted cofactor mixture, 2.1 mL reconstituted enzyme mixture, 2.3 mL ddH_2_O, and 0.45 mL reconstituted DTNB. The plate cover was then removed and 150 μL of the freshly prepared assay cocktail was added to each well. The cover was replaced, and the plate was incubated in the dark on a shaker for 25 min. The absorbance at 405 nm was assessed with the Benchmark Plus Microplate Reader (Bio-Rad, Hercules, CA, USA). The GSH levels in plasma were determined using a standard curve, and all GSH analyses were repeated twice.

### 2.4. Statistical Analysis

All subjects’ clinical characteristics and GSH levels were presented as the mean (SD) or number (percentage). We compared mean values between two groups by using the Mann–Whitney U test and percentages using the χ^2^ test. 

The GSH levels and cognitive function assessments (MMSE and ADAS-cog scores) at baseline and at endpoint (at 2 years later) were compared by paired *t* test. 

Multiple linear regressions were used to discover independent factors associated with cognitive change in MCI patients (stepwise). 

All analyses were performed using IBM SPSS, version 20 (IBM Corp., Armonk, NY, USA), and a two-tailed *p* < 0.05 was considered statistically significant.

## 3. Results

Overall, 65 subjects were enrolled, and they included 16 healthy individuals and 49 patients with MCI. The demographic, clinical, and laboratory data of both groups at baseline are summarized in Table 1.

Gender (female 81.3% in the healthy group vs. 69.3% in the MCI group) and age (68.9 ± 5.9 years vs. 69.9 ± 8.4 years) distributions were similar in the two groups. Female gender was the predominant gender of both groups. Compared to healthy individuals, MCI patients had shorter education duration (9.6 (mean) ± 2.7 (SD) vs. 6.7 ± 4.4, *p* = 0.003), lower MMSE score (28.7 ± 1.1 vs. 25.3 ± 3.8, *p* < 0.001), and higher ADAS-cog score (3.5 ± 1.7 vs. 9.4 ± 7.1, *p* = 0.001). As to plasma GSH, the healthy individuals and the MCI patients had similar levels (4.1 ± 2.7 vs. 4.1 ± 2.5 ng/mL, respectively).

### 3.1. GSH Levels and Cognitive Function Differed Significantly between Baseline and Endpoint among MCI Patients but Not among Healthy Controls

Table 2 displays the changes in GSH concentration and cognitive function during the two-year follow up period. At first, the healthy elderly’s GSH concentration (4.1 ± 2.7 to 3.0 ± 3.1 ng/mL) and cognitive function (in terms of both ADAS-cog (3.5 ± 1.7 to 3.1 ± 1.6 and MMSE (28.7 ± 1.1 to 28.3 ± 1.2) scores) did not change significantly from baseline to endpoint (24 months later). However, GSH levels appeared lower (1.5 ± 1.3 ng/mL) at 18 months.

In comparison, the MCI patients showed significant decline in plasma GSH levels (4.1 ± 2.5 to 2.2 ± 2.7 ng/mL, *p* < 0.001) and cognitive function (in terms of both ADAS-cog (9.4 ± 7.1 to 11.1 ± 9.1, *p* = 0.035) and MMSE (25.3 ± 3.8 to 24.0 ± 4.1, *p* = 0.006) scores) from baseline to endpoint.

### 3.2. Both GSH Level at Baseline and GSH Level Changed from Baseline to Endpoint Significantly Influenced Cognitive Decline over 2 Years among the MCI Patients 

We then tested whether (1) GSH concentration at baseline and (2) GSH concentration changed from baseline to endpoint (endpoint–baseline) were able to affect cognitive decline during the two-year period among the MCI patients, respectively. 

Firstly, Table 3 presents multiple linear regression analyses of independent factors (including baseline GSH level, age, gender, and education duration) associated with cognitive change from baseline to endpoint in MCI patients (stepwise). The results showed that GSH concentration at baseline was positively associated with ADAS-cog score change from baseline to endpoint (month 24). Other factors (age, gender, and education duration) didn’t significantly influence cognitive change, though education showed a trend (*p* = 0.068). That is, higher GSH concentration at baseline was associated with more cognitive decline at endpoint among the MCI patients.

Secondly, Table 4 presents multiple linear regression analyses of independent factors (including GSH level change from baseline to endpoint, age, gender, education duration) associated with cognitive change from baseline to endpoint in MCI patients (stepwise). The results showed that female (vs. male) and GSH level change were negatively associated with ADAS-cog score change from baseline to endpoint. Other factors (age and education duration) didn’t significantly influence cognitive change, though education showed a trend (*p* = 0.067). That is, female and higher GSH level changes from baseline to endpoint were associated with better cognitive outcome at endpoint among the MCI patients.

## 4. Discussion

To the best of our knowledge, this was the first study to explore the role of GSH concentration in very early-phase AD (herein MCI), and the first one to prospectively follow GSH levels during the AD-related illness course. 

In accordance with the findings from previous AD patients or non-demented individuals [15,17,18,19,22], the current study demonstrated that both plasma GSH levels (from 4.1 ± 2.5 at baseline to 2.2 ± 2.7 ng/mL at endpoint (*p* < 0.001)) and cognitive function (mean ADAS-cog score from 9.4 ± 7.1 to 11.1 ± 9.1 (*p* = 0.035); and MMSE from 25.3 ± 3.8 to 24.0 ± 4.1 (*p* = 0.006)) declined 2 years later among the patients with MCI (Table 2). In contrast, among the healthy elderly, the GSH levels, albeit with fluctuation, did not change significantly between baseline (4.1 ± 2.7 ng/mL) and endpoint (3.0 ± 3.1 ng/mL), while their cognitive function also remained stable (ADAS-cog score from 3.5 ± 1.7 to 3.1 ± 1.6; MMSE from 28.7 ± 1.1 to 28.3 ± 1.2) (Table 2). These findings suggest the importance of periodically monitoring GSH concentration as well as cognitive function for the MCI patients in not only clinical service but also clinical trials. Moreover, the findings could be the basis for setting-up precision medicine in the field of cognitive aging in the future.

Another main finding is that both GSH level at baseline and GSH level change from baseline to endpoint significantly influenced cognitive decline over 2 years among the MCI patients by multiple linear regression analyses (stepwise) (Table 3 and Table 4), thereby implying the role of GSH in modulating cognitive function and further emphasizing the importance of monitoring GSH levels among the MCI patients. Likewise, previous studies showed the role of blood GSH concentration in regulating cognitive function of AD patients [20] and in preserving executive function of non-demented people [22]. 

The etiology of the gradual decline in GSH levels during the 2 years MCI period (Table 2) remains unclear. GSH synthesis is regulated by the cystine/glutamate antiporter system x_c_^–^ [29], which is a sodium-independent acidic amino acid transporter mediating the uptake of cystine into cells in exchange for glutamate in a 1:1 ratio [30]. Cystine is reduced to cysteine intracellularly after its incorporation into system x_c_^–^ and cysteine is the rate-limiting substrate in the biosynthesis of GSH [16]. Of note, x_c_^–^ inhibition attenuates GSH synthesis, perturbing cellular redox balance [31]. It has been shown that peripheral expression of system x_c_^–^ was lower in patients with other neurodegenerative disorders, such as schizophrenia, than healthy controls [32,33]. Since plasma GSH levels may gradually diminish during the MCI phase, whether the activity or expression of system x_c_^–^ also gradually reduce during the very-early AD phase deserves study in the future.

In addition, it has been well known that education is a protective factor of cognitive aging [34]. In accordance, in the current study, compared to healthy individuals, MCI patients had shorter mean education duration (9.6 ± 2.7 years vs. 6.7 ± 4.4 years, *p* = 0.003), while the healthy individuals and the MCI patients had similar GSH levels (4.1 ± 2.7 ng/mL vs. 4.1 ± 2.5 ng/mL, respectively) (Table 1). The finding suggests that the elderly people who have lower education levels (albeit with similar GSH concentrations) may be more likely to have impaired cognitive function. Moreover, though education duration did not significantly protect cognitive change during the two-year follow-up period, it showed a trend (*p* = 0.068 in Table 3; 0.067 in Table 4, by multiple regression analyses). 

Finally, being female appeared to be another protective factor of cognitive decline, especially with controlling GSH level change from baseline to endpoint (*p* = 0.040 in Table 4). Similarly, a recent study found that being female was a marginal protective factor of cognitive function in aging after memory training [35]. However, in another study, women with MCI were more likely to decline in cognitive function than men [36]. More studies are needed to elucidate the role of gender in MCI progression.

### 4.1. Strength and Limitation

The major strength of the current study is that we examined both cross-sectional and longitudinal effects of GSH levels in cognitive function in the MCI patients. Second, we employed not only MMSE [27] but also ADAS-cog [26] (which can provide more comprehensive cognitive evaluation) in this prospective study.

There are also limitations in the present study. First, our study did not evaluate other endogenous antioxidants, which may also reduce oxidative damage in the cognitive aging process [37]. Second, the peripheral blood–CNS relationship of GSH requires investigation in patients with MCI. Third, the participants in this study were ethnic Han Chinese cohort. Our study findings may not be generalizable to other populations. Fourth, the number of subjects was small, and the follow-up duration was modest. Fifth, GSH levels can be influenced by environmental factors such as diet [38]. In our study, we did not control or measure subjects’ diet, which may have contributed to the unstable GSH levels (particularly at month 18) in healthy subjects. Future studies which control or measure subjects’ diet or nutrients are helpful. 

### 4.2. Clinical Implication and Future Direction

MCI represents the transitional state between healthy aging and dementia [8]. Therefore, it is important to discover a feasible blood biomarker for predicting and monitoring the disease course. In the current study, the findings suggest that peripheral GSH concentration may be a good marker in assisting physicians in predicting and monitoring of early cognitive aging. 

Further studies with large sample sizes and longer duration are warranted to adequately elucidate the role of GSH in cognitive aging.

## 5. Conclusions

This study demonstrated that both plasma GSH levels and cognitive function declined 2 years later, and that both GSH level at baseline and GSH level change from baseline to endpoint significantly influenced cognitive decline over 2 years among the MCI patients. Nevertheless, further studies with a larger sample size and longer follow-up duration are required to evaluate the temporal relationship between GSH levels and the MCI progression. If replicated by future studies, blood GSH concentration may be regarded as a biomarker for monitoring cognitive change in MCI.

## Figures and Tables

**Table 1:** Demographic characteristics of the overall cohort (*n* = 65) at baseline.

	Healthy Elderly (*n* = 16)	MCI Patients (*n* = 49)	*p*
Demographics			
CDR, mean (SD)	0	0.5	
Gender, female, *n* (%)	13 (81.3)	33 (67.3)	0.357 ^a^
Age, year, mean (SD)	68.9 (5.9)	69.9 (8.4)	0.314 ^b^
Education, year, mean (SD)	9.6 (2.7)	6.7 (4.4)	0.003 ^b^
MMSE, mean (SD)	28.7 (1.1)	25.3 (3.8)	<0.001 ^b^
ADAS-cog, mean (SD)	3.5 (1.7)	9.4 (7.1)	0.001 ^b^
GSH level, ng/mL, mean (SD)	4.1 (2.7)	4.1 (2.5)	0.726 ^b^

^a^ Chi-square test; ^b^ Mann–Whitney U test; MCI, mild cognitive impairment; CDR, clinical dementia rating; MMSE, Mini-Mental Status Examination; ADAS-cog, the Alzheimer’s disease assessment scale-cognitive subscale; GSH, glutathione.

**Table 2:** The changes in GSH level and cognitive function during the 2 years period.

Healthy Elderly (*n* = 16)			
Variable	GSH Level, ng/mL	ADAS-Cog	MMSE
Baseline	4.1 (2.7)	3.5 (1.7)	28.7 (1.1)
6 months	4.1 (3.3)	3.2 (1.2)	28.6 (1.5)
12 months	2.9 (2.9)	3.4 (2.1)	28.1 (1.0)
18 months	1.5 (1.3)	3.1 (1.4)	28.3 (1.1)
24 months	3.0 (3.1)	3.1 (1.6)	28.3 (1.2)
*p*-Value of paired *t*-test (Baseline vs. 24 months)	0.053	0.302	0.234
MCI patients (*n* = 49)			
Variable	GSH level (ng/mL)	ADAS-cog	MMSE
Baseline	4.1 (2.5)	9.4 (7.1)	25.3 (3.8)
6 months	3.5 (3.8)	8.7 (6.0)	25.6 (3.2)
12 months	2.8 (2.8)	9.3 (6.4)	25.0 (3.9)
18 months	2.7 (2.3)	10.5 (8.3)	24.3 (4.3)
24 months	2.2 (2.7)	11.1 (9.1)	24.0 (4.1)
*p*-Value of paired *t*-test (Baseline vs. 24 months)	<0.001	0.035	0.006

GSH, glutathione; ADAS-cog, the Alzheimer’s disease assessment scale-cognitive subscale; MMSE, Mini-Mental Status Examination; MCI, mild cognitive impairment.

**Table 3:** Multiple linear regression analyses of independent factors (including baseline GSH level) associated with cognitive change, measured by ADAS-cog score change from baseline to endpoint at month 24, in MCI patients (stepwise).

MCI Patients (*n* = 49)			
Variable	B (SE)	t	*p*
Gender, female vs. male	−2.683 (1.556)	−1.724	0.091
Education, year	−0.309 (0.166)	−1.867	0.068
Baseline GSH level, ng/mL	0.882 (0.280)	3.149	0.003
Adjusted R square = 0.227			

The regression model was adjusted with age, gender, education, and baseline GSH level. Significant variables are shown as *p* < 0.05. Abbreviations: GSH, glutathione; ADAS-cog, the Alzheimer’s Disease Assessment Scale-Cognitive Subscale; MCI, mild cognitive impairment.

**Table 4:** Multiple linear regression analyses of independent factors (including GSH level change from baseline to endpoint) associated with cognitive change, measured by ADAS-cog score change from baseline to endpoint, in MCI patients (stepwise).

MCI (*n* = 49)			
Variable	B (SE)	t	*p*
Gender, female vs. male	−3.306 (1.566)	−2.110	0.040
Education, year	−0.316 (0.169)	−1.875	0.067
GSH level change, ng/mL	−0.621 (0.222)	−2.796	0.008
Adjusted R square = 0.196			

The regression model was adjusted with age, gender, education, and GSH level change. Significant variables are shown as *p* < 0.05. Abbreviations: GSH, glutathione; ADAS-cog, the Alzheimer’s Disease Assessment Scale-Cognitive Subscale; MCI, mild cognitive impairment.

## Data Availability

Data is contained within the article.
